# New Methods for Imaging Evaluation of Chest Wall Deformities

**DOI:** 10.3389/fped.2017.00257

**Published:** 2017-12-04

**Authors:** Ana Lain, Laura Garcia, Carlos Gine, Olivier Tiffet, Manuel Lopez

**Affiliations:** ^1^Department of Pediatric Surgery, University Hospital of Vall d’Hebron, Universidad Autonoma de Barcelona, Barcelona, Spain; ^2^Department of Adults General Surgery and Thoracic Surgery, University Hospital of Saint-Étienne, Saint-Étienne, France

**Keywords:** pectus carinatum, pectus excavatum, noninvasive evaluation, free irradiation, objectives assessment

## Abstract

**Aim:**

The purpose of this study is to describe the development of an external 3-dimensional (3D) scanner as a noninvasive method for imaging chest wall deformities. It allows objective assessment, reconstruction of the area of interest, and evaluation of the severity of the deformity by using external indexes.

**External 3D scanning system:**

The OrtenBodyOne scanner (Orten, Lyon, France) uses depth sensors to scan the entire 3D external body surface of a patient. The depth sensors combine structured light with two classic computer vision techniques: depth from focus and depth from stereo. The data acquired are processed and analyzed using the Orten-Clinic software.

**Materials and methods:**

To investigate the performance of the device, a preliminary prospective study (January 2015–March 2016) was carried out in patients attending our hospital chest wall deformities unit. In total, 100 patients (children and young adults) with pectus excavatum or pectus carinatum, treated by surgery or non-operative methods were included. In patients undergoing non-operative treatment, external 3D scanning was performed monthly until complete correction was achieved. In surgically treated patients, scanning was done before and after surgical correction. In 42 patients, computed tomography (CT) was additionally performed and correlations between the Haller index calculated by CT and the external Haller index using external scanning were investigated using a Student’s test (*r* = 0.83).

**Conclusion:**

External scanning is an effective, objective, radiation-free means to diagnose and follow-up patients with chest wall deformities. Externally measured indexes can be used to evaluate the severity of these conditions and the treatment outcomes.

## Introduction

Pectus excavatum (PE) and pectus carinatum (PC) are the most common congenital chest wall defects. PE or sunken chest is characterized by posterior depression of the sternum (1). This defect may be associated with impaired cardiac and pulmonary function, resulting in a decreased ability for patients to perform vigorous cardiovascular activity relative to their peers. A secondary effect of the deformity is the socially limiting psychological stress it can cause (2). PC or pigeon chest results from excessive growth of the costal cartilage (3), which typically results in protrusion of the sternum. In contrast to PE, clothing does not conceal PC. PC is mainly a cosmetic problem, although it can be psychologically distressing, with patients having a negative self-image and poorer quality of life than individuals without this deformity.

In addition to radiography, computed tomography (CT)imaging is one of the standard methods used to evaluate the extent of chest wall deformities. In PE, CT is often used to calculate the Haller index (HI) (4), a metric to determine the severity of the deformity. Nevertheless, radiography and CT examinations imply radiation exposure, and in pediatric patients, every effort should be made to limit radiation. Children should undergo a CT examination only when it is essential, and they should not have repeated CT studies unless absolutely necessary.

Objective methods to evaluate the effectiveness of surgical and non-operative treatments for pectus deformities are limited, and again, radiographies or CT scans are often required. The use of these techniques for treatment follow-up may be reserved for the most severe cases, leaving many patients with minor or moderate deformities without assessment. Magnetic resonance imaging (MRI) can also be used for this purpose, but it takes longer to acquire the images and is a costly technique for repeated examinations.

After the introduction of non-operative treatments for chest wall deformities, the evaluation methods described in the literature have been mainly qualitative, such as medical photography, satisfaction scoring scales, and measurement tools to evaluate the deepest point in cases of PE and the highest point in PC (5–8). It would be of considerable value to have a more objective, noninvasive method for assessing chest wall deformities to facilitate the treatment decision process and post-treatment follow-up. The purpose of this study is to describe the development of a 3-dimensional (3D) external scanning technique presented as a novel, noninvasive imaging method to evaluate chest wall deformities.

## Materials and Methods

### External 3D Scanning System

**Figure 1:**
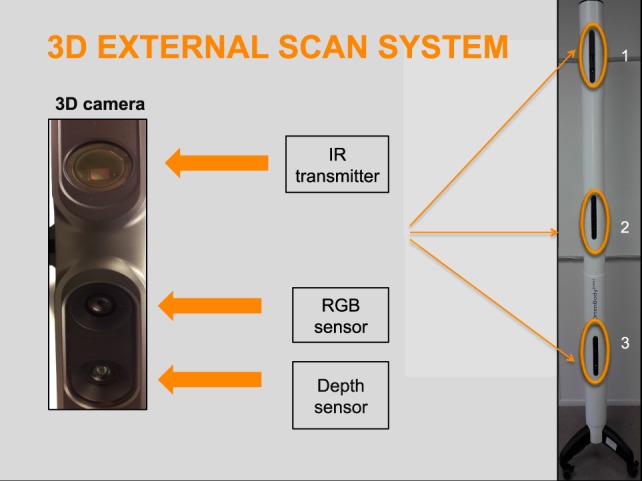
External 3-dimensional (3D) scanning system. IR, infrared; RGB, Red-green-blue.

The OrtenBodyOne external 3D scanner has been developed within a collaborative effort between Vall d’Hebron Hospital and the Orten clinical research society (Lyon, France). The system is composed of a scanner column and a turntable with vertical bars. The scanner uses 3D depth sensors to acquire data on the entire external 3D body surface topography of the patient. These depth sensors combine structured light with two classic computer vision techniques: depth from focus and depth from stereo. The first sensor uses infrared laser lighting with a speckle pattern to create the 3D shape. The second, red-green-blue, sensor detects and displays images to an electronic system and projects texture and color on the 3D surface mesh. The third depth sensor is used to construct the image by analyzing the speckle pattern of infrared laser light. The infrared and depth sensors set the data on an area (Figure 1).

Optical imaging techniques are able to optically assess the surface appearance and develop a 3D surface model with superimposed texture maps of patients with chest wall deformities without the use of radiation (9). They enable noninvasive scanning of children, adolescents, and adults.

#### Data Acquisition

**Figure 2:**
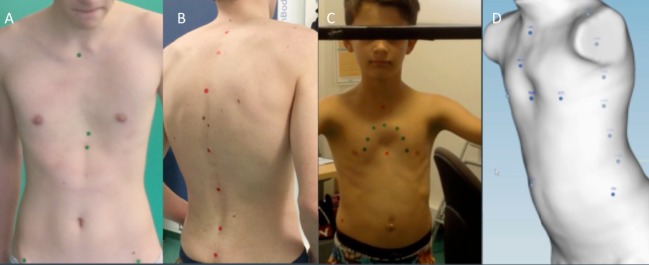
Anatomic landmarks. Landmarks placed in the front **(A)** and back **(B)** of the trunk. Additional landmarks in Currarino Silverman Syndrome **(C,D)**.

Prior to data acquisition, a trained medical operator locates strategic anatomical landmarks on the patient’s trunk by palpation and places markers on them. These markers relate internal bone structures with the external surface landmarks. The five landmarks placed on the anterior trunk include the sternal notch, highest or deepest point of the deformity, xiphoid process, and left and right anterior superior iliac spines. On the posterior trunk, seven landmarks are placed at the spinous apophyses from C7 to S1. Additional landmarks can be added in atypical deformities, such as Currarino Silverman syndrome (Figure 2).

#### Patient Positioning and Acquisition Time

Patients stand on the turntable with their hands on the bars. The hands are placed at shoulder level to achieve a standardized position during data acquisition. The procedure takes around 24 s, corresponding to the time needed for one complete lap on the turntable. The three views from the depth sensors merge to create a 3D surface rendering of the body.

#### Data Processing

The reconstruction time takes about 4 min. The previously placed anatomical landmarks are manually identified during data processing. The first step is to erase all parts of the body that are not within the area of interest to be analyzed. The lower and upper portions of the torso are erased at the pants line and neck level, respectively, and the arms are erased through the corners of the acromion. In a very few cases, the depth sensors may not properly capture some small surfaces (e.g., the top of the shoulders). These areas can create holes and spikes in the trunk model. In these cases, the surface is re-meshed using the hole-filling tool to correct the scanning artifact and obtain a smooth, closed surface.

### Pectus Analysis

The data obtained are processed by Orten-Clinic software (Orten, Lyon, France) to obtain a 3D reconstruction of the area of interest, which can be used to derive various external measures and indices. The features are defined by the pectus landmarks placed previously. Two types of pectus deformity analyses have been developed with this software: initial and longitudinal.

#### Initial Analysis

In the initial simple analysis, the configuration and severity of the deformity are evaluated using several measurements and external indexes from a single examination. The trunk surface topography is created by 3D reconstruction. A cross-sectional image of the trunk is taken at the level of the deepest point in PE, the highest point in PC, or at both points in mixed PE/PC deformities.

In PC patients who are candidates for non-operative treatment with dynamic compression systems, the software performs automatic chest measurements so that the brace can be customized without the need for manual measurement during the physical examination.

At the same time, the software evaluates any potential musculoskeletal abnormalities, such as postural and spinal abnormalities, with detection of varying degrees of scoliosis in some cases.

The following measurements can be obtained in patients with PE or PC.

The distance between the

– pectus point and the point of the theoretical anatomical shape,
– pectus point and spinal line point,
– pectus point and back point (obtained in the sagittal plane),
– angles of the spinal line and of the sternal line,
– circumference line of the transversal section.

#### Longitudinal Analysis

**Figure 3:**
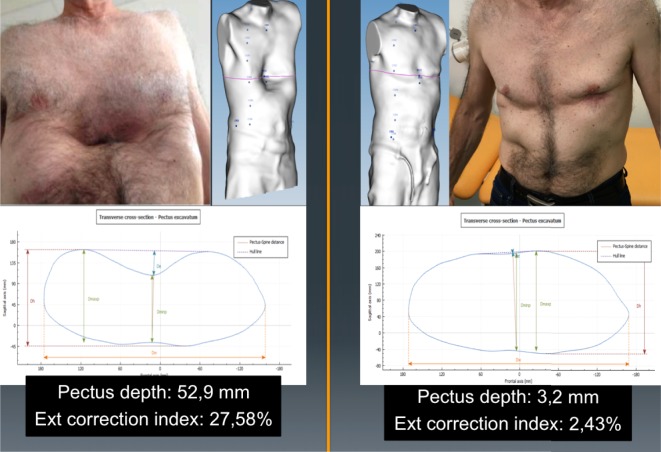
Longitudinal analysis. External correction index in the same patient before and after the Nuss procedure. De, deepest point; Dmaxp, maximum length; Dminp, minimum length; Hull line, perimeter.

Longitudinal analysis can be used to monitor the changes in the chest wall defect during non-operative treatment. Images obtained in serial scans can be superimposed to compare the related measures and indexes over time. The changes occurring between several external 3D scans can therefore be objectively assessed to determine the effectiveness or failure of non-operative treatment. Additionally, it can be used to compare the patient’s status before and after surgical correction (Figure 3).

### Relevant Clinical Parameters

#### Pectus Excavatum

##### Deepest Point (De)

**Figure 4:**
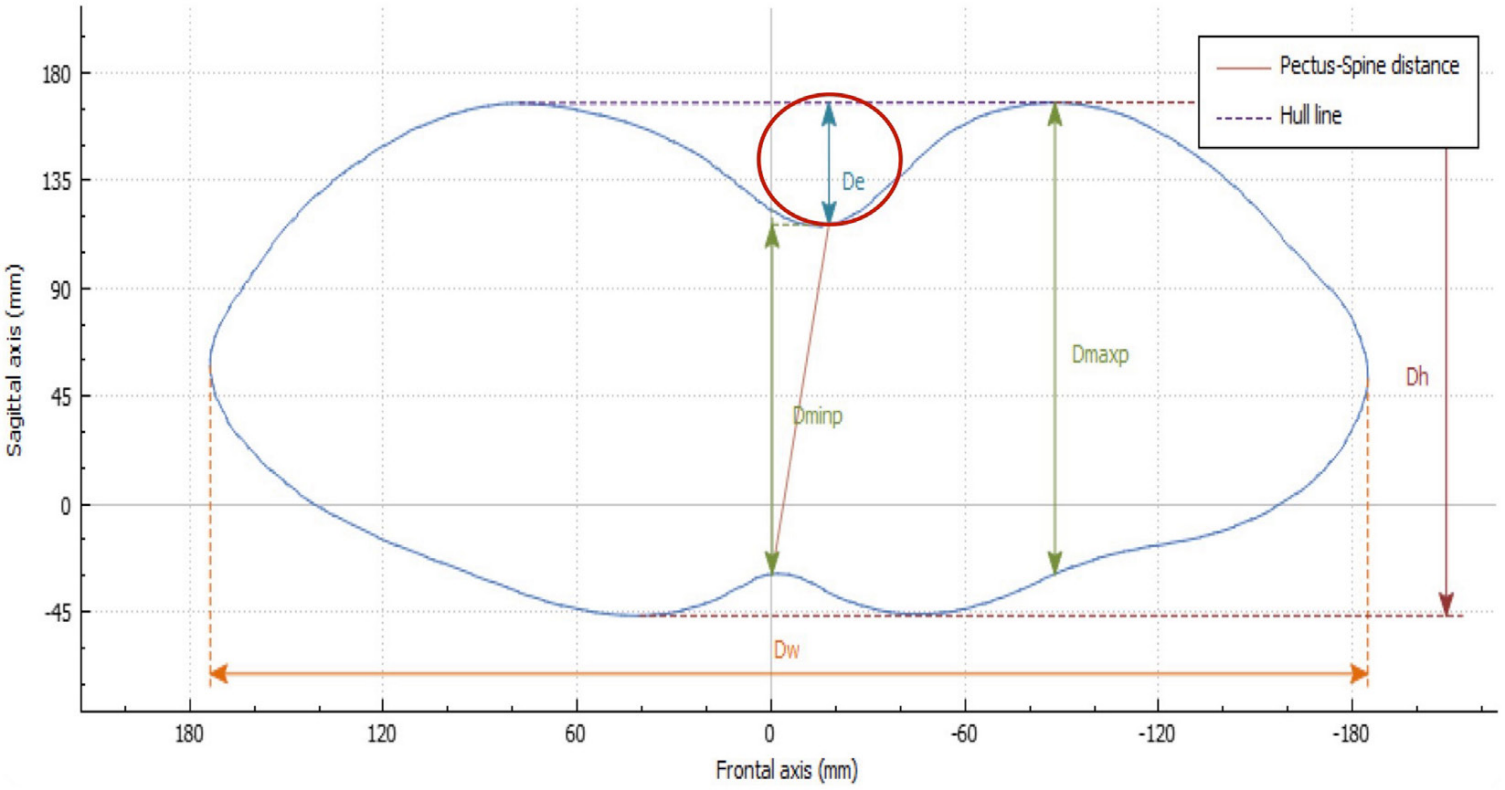
Deepest point. The deepest point is the difference between the maximum and the minimum diameter (Dmaxp − Dminp). De, deepest point; Dmaxp, maximum length; Dminp, minimum length; Dh, anterior–posterior distance; DW, horizontal external distance; Hull line, perimeter.

**Figure 5:**
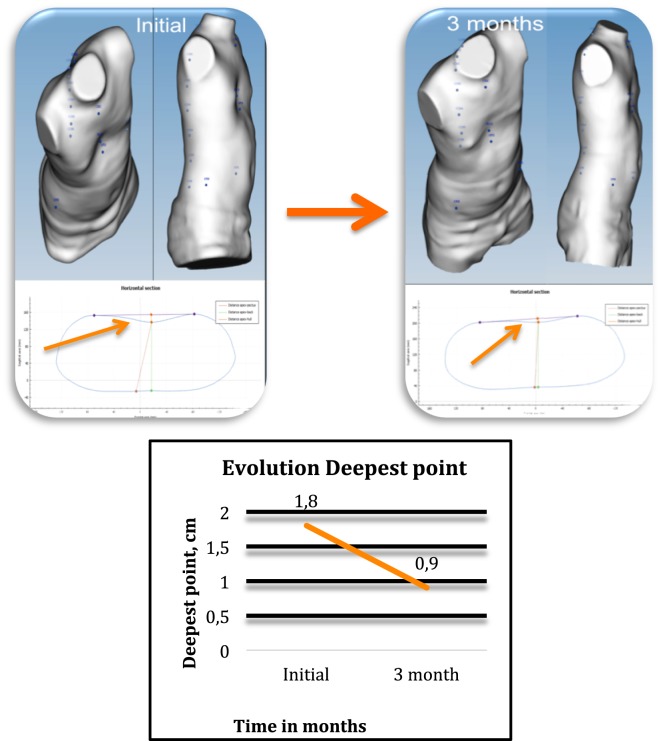
Changes occurring in the deepest point in non-operative treatment. External 3-dimensional imaging of the same pectus excavatum patient before and at 3 months after starting cup suction treatment with a vacuum bell (4 h/day), showing changes in the deepest point.

De is calculated as the depth of the skin surface at the level of the deepest point. It is the difference between maximal to minimal diameter (Dmaxp − Dminp) in the deepest point of the malformation (Figure 4). This measure can be used to assess the efficacy of non-operative treatment in PE (e.g., vacuum bell) (Figure 5). To avoid bias, a second measurement is taken at the level of the deepest point between the spinal line point and deepest pectus point.

##### External Haller Index (EHI)

**Figure 6:**
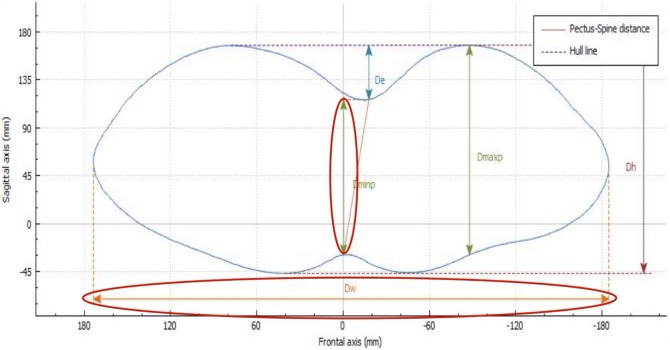
External Haller index (EHI). The EHI is the lateral external distance divided by the distance between the external deepest point and external spinous process (Dw/Dminp). De, deepest point; Dmaxp, maximum length; Dminp, minimum length; Dh, anterior–posterior distance; DW, horizontal external distance; Hull line, perimeter.

The HI is the standard severity index used to evaluate PE with radiography, CT imaging, or MRI. It is dependent on the width of the thorax and, at times, may not properly assess the depth of the defect. With use of the 3D scanner, an equivalent index is obtained: the EHI. From the optical cross section of the torso, reconstruction measures are taken from the posterior portion of the vertebral bodies. The EHI is the lateral external distance divided by the distance between the external deepest point and external spinous process (Dw/Dminp) (Figure 6).

##### External Correction Index (ECI)

Another index, the standard correction index (CI), has been found to more perfectly separate normal individuals and the patient population with PE than the traditional HI (10). The original CI requires drawing a horizontal line across the anterior spine. Two distances are then measured: the minimum distance between the posterior sternum and anterior spine and the maximum distance between the line placed on the anterior spine and the inner margin of the most anterior portion of the chest. The difference between the two lines is simply the magnitude of the chest defect.

If this difference between the two measurements is then divided by the maximum prominence of the chest (the longer measurement) and multiplied by 100, it generates the percentage of chest depth the patient is missing centrally. Using external scanning, the ECI can be easily and reproducibly calculated using the skin surface measures obtained.

##### External Sternal Depression Angle: “Banana Index”

**Figure 7:**
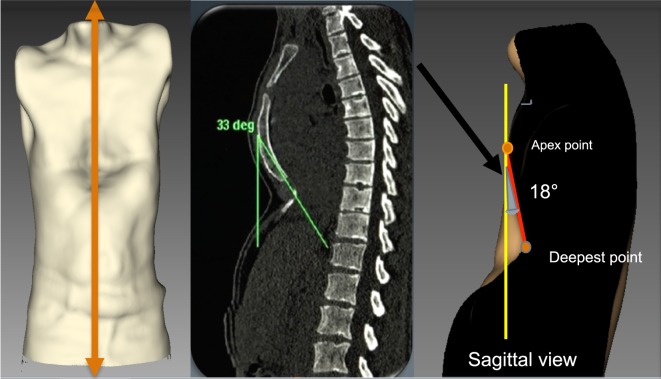
External angle of sternal depression “Banana index.” The orange line indicates the sagittal section through the sternum. The angle is defined between the red line and the yellow line. The gray shaded area defined by the red line and yellow line is the banana index.

The purpose of this index is to evaluate the angle of the sternal depression. To calculate the index, it is important that the anterior anatomic landmarks (sternal notch, deepest point, and xiphoid process) are correctly placed prior to data acquisition with the 3D scanner. Using the image analyzer, a sagittal cross section is obtained through the sternum at the midline of the trunk, allowing evaluation the total sternal surface (Figure 7). The angle between the theoretical anatomical sternal line and the sternal line of the patient is the “banana index.” The larger the angle, the greater the depression.

This index can be used to plan strategies for corrective PE surgery and for localizing the best positions to place the Nuss bars.

##### External “Titanic Index”

**Figure 8:**
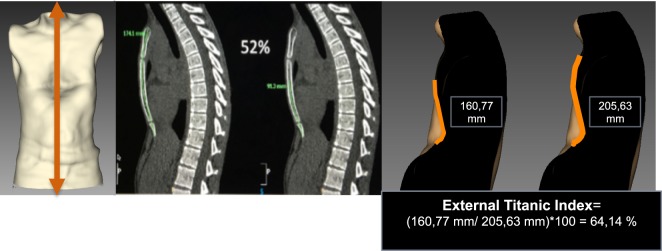
External Titanic index in pectus excavatum. A cross section is obtained through the sternum at the midline of the trunk. The sagittal section of 3-dimensional shape allows evaluating the percentage of depressed sternal length: ((depressed sternal length/complete sternal length) × 100).

The Titanic index (TI), that is, the percentage of “sunken” sternum, has been described by Martinez-Ferro using measures obtained from standard CT scanning. To calculate the corresponding external index, anterior anatomic landmarks should be placed at the sternal notch, the deepest point, and the xiphoid process. Using the image analyzer, a sagittal cross section is obtained through the sternum at the midline of the trunk. The sagittal section of the 3D shape allows evaluating the percentage of depressed sternal length: (depressed sternal length/complete sternal length) × 100. This measure helps to decide the number of correction bars the patient will need (Figure 8).

#### Pectus Carinatum

##### Highest Point

**Figure 9:**
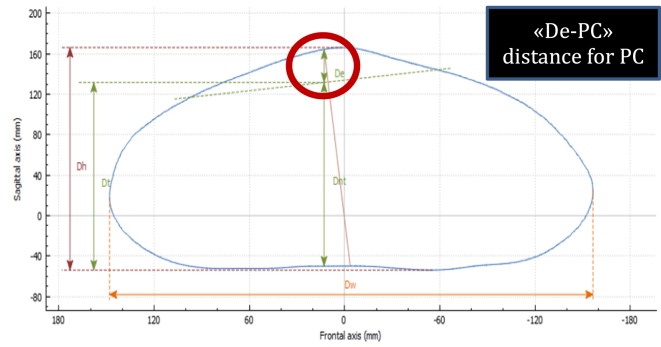
Highest point in pectus carinatum (PC). The highest point is calculated by delineating a cross section at the level of the highest point and another at the nipples. The difference between the two measures is the height of the deformity. De-PC, highest point in PC; Dh, anterior–posterior distance; Dnt, distance from the spinous process to the nipples; Dt, distance from horizontal posterior line to the nipples; Dw, horizontal external distance.

The highest point is calculated by placing landmarks at the highest point of the protrusion and the nipples. A perpendicular line is drawn at the level of the highest point and another at the nipples. The difference between the two measures is the height of the deformity (Figure 9).

**Figure 10:**
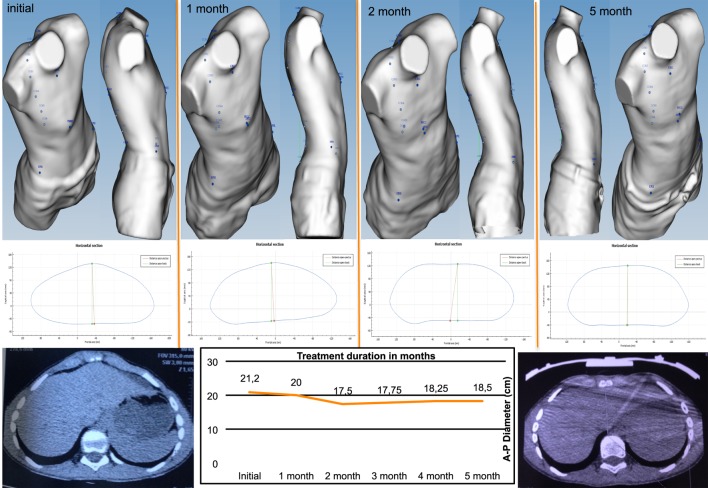
Changes occurring in the highest point in non-operative treatment of pectus carinatum. Longitudinal analysis: images can be superimposed, and changes occurring between several 3-dimensional scans can be compared to determine whether non-operative dynamic compression treatment is successful.

External 3D scanning can be performed monthly in PC patients treated by dynamic compression to monitor the effectiveness of the treatment. In this type of longitudinal analysis, the images acquired can be superimposed to compare the changes occurring in the deformity over time. Additionally, it can be used to compare the status of the deformity before and after surgical correction (Figure 10).

##### External Haller Index

The EHI can also be used to assess the degree of sternal protrusion in PC, where the anterior–posterior diameter is measured from the surface of the sternum (point of maximal protrusion) to the posterior vertebral body.

##### Asymmetry Analysis

**Figure 11:**
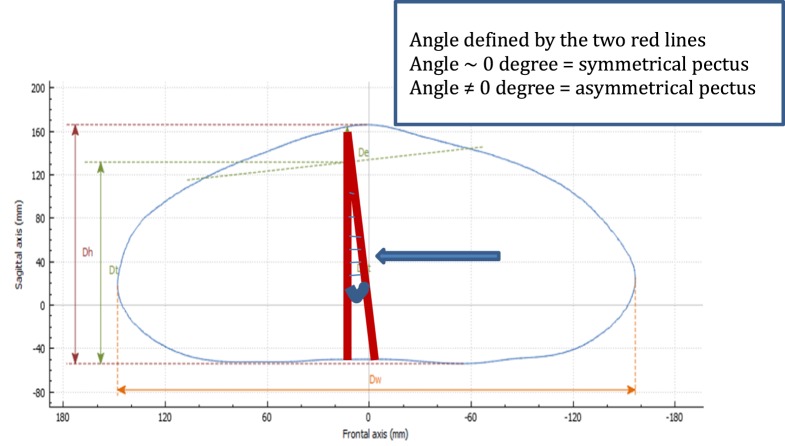
Asymmetry analysis in pectus carinatum. Two lines are drawn, one from the highest point to the center of the horizontal line (Dw), and a perpendicular line from the horizontal plane to the highest point. In cases of asymmetry, the angle will be other than 0°.

Determines whether the deformity is asymmetrical or not. Two lines are drawn from the highest point of the anterior deformity, one perpendicular to the back line and another to the center of the back. The angle between these two lines indicates the symmetry of the deformity. In cases of asymmetry, the angle will have a value other than 0° (Figure 11).

##### Pectus Balance in Mixed Deformities

This software feature shows whether the volumes of the body are well distributed on the right and left. With the use of colorimetric analysis, the distribution of volumes on the two sides of the chest can be compared. In patients with mixed abnormalities (PC plus PE), the treatment approach can be a challenge, particularly in patients undergoing non-operative treatment with dynamic compression for PC and cup suction for PE. The doubt arises as to which should be treated first.

**Figure 12:**
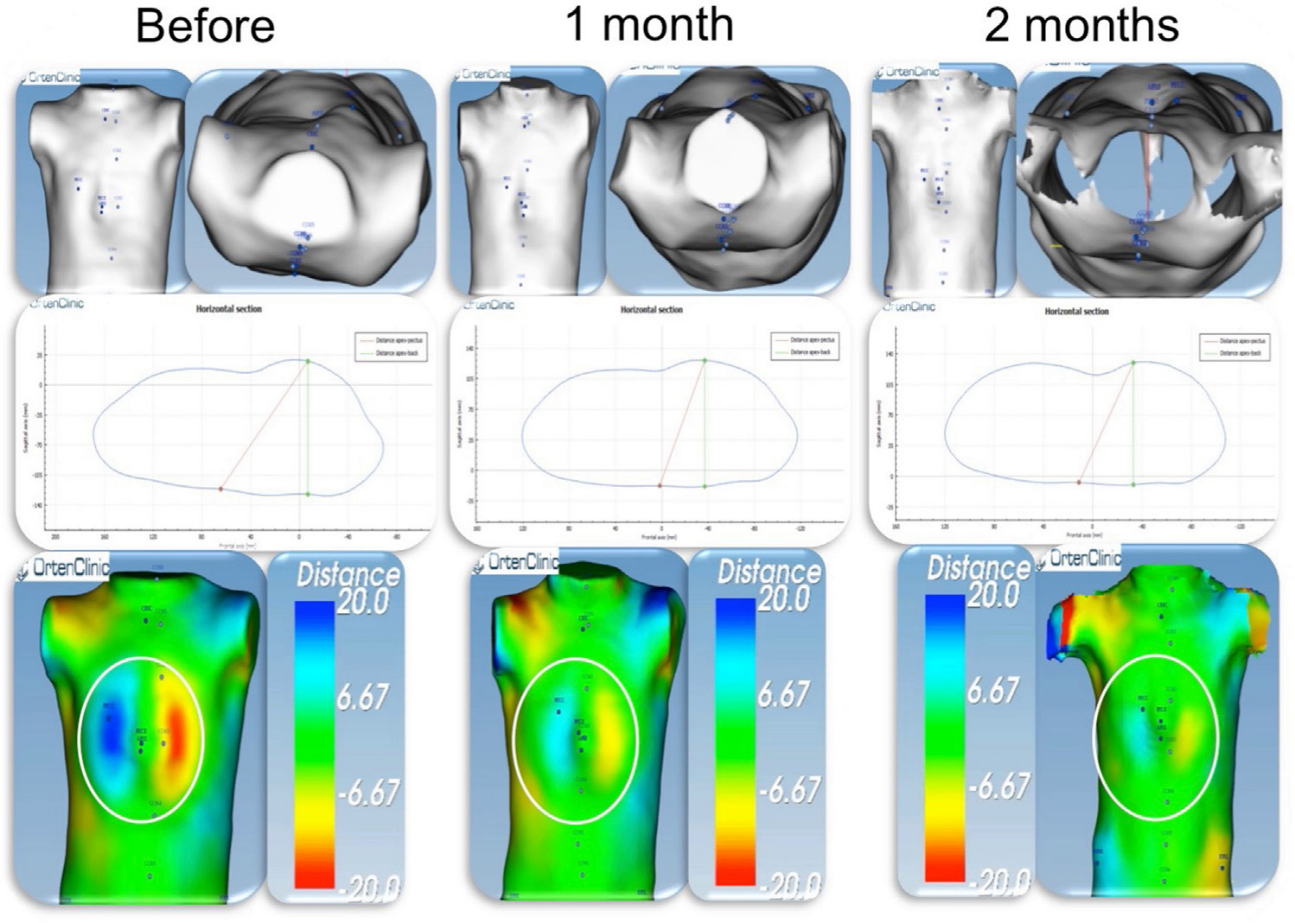
Pectus balance in a patient with mixed deformity (PC + PE). Colorimetric study. External scan of a patient with mixed deformity, PC on the right side (blue), and PE on the left side (red). Before starting PC treatment dynamic compression, the distance was around 40 mm. After 2 months of treatment, the 2 sides are almost symmetrical (green on both sides). At this time point, PC treatment was switched to cup suction for PE. PC, pectus carinatum; PE, pectus excavatum.

Based on our experience, we believe that the PC component of the deformity should be treated first until a symmetrical deformity is achieved, and then treatment should be switched to cup suction for PE. The use of colorimetric analysis enables objective evaluation of the status of the deformity to determine the optimal time to switch treatment (Figure 12).

### Posture and Spine Evaluation

In previous published series of patients with chest wall deformities, 90% had associated postural problems and 23% had scoliosis (7, 8). In most patients, physiotherapy was added to the corrective treatment to optimize the results.

**Figure 13:**
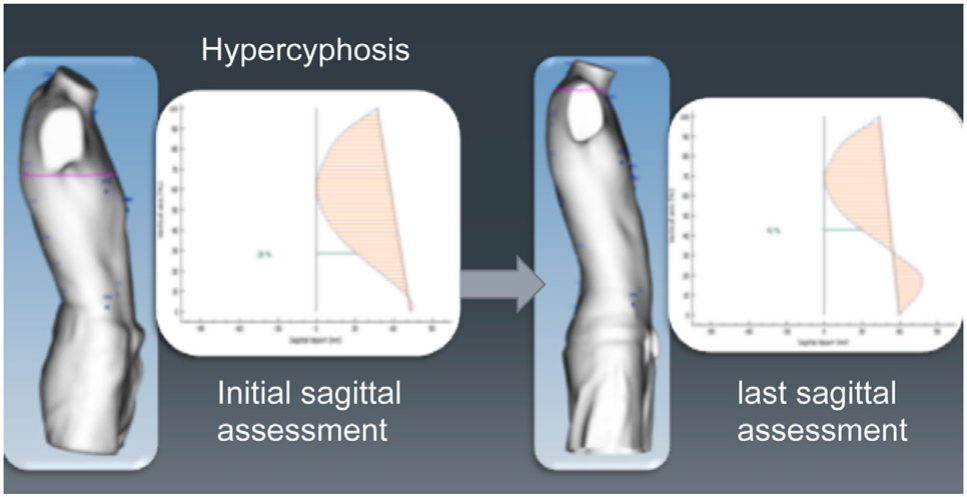
Evaluation of abnormal posture using external 3-dimensional scanning. Same patient before and during non-operative treatment, showing an improvement in his posture.

To determine back anomalies, analysis of the sagittal spine curve is carried out by automatic detection of the anatomical landmarks placed by the operator between C7 and S1. The spinous process line should be assessed in the sagittal plane to evaluate back posture, a relevant parameter in chest wall deformities. Balance between the kyphosis area and lordosis area indicates good posture. Conversely, a lack of balance indicates a postural anomaly marked by hyper-kyphosis relative to lordosis (Figure 13).

### Statistical Analysis

#### Methods

A prospective study was conducted from January 2015 to March 2016 and a preliminary evaluation of the performance of the technique was carried out. All patients with chest wall deformities consulting at our center were assessed for surgical correction or non-operative treatment. The study was approved by the local ethics review board, and patients or their families provided written consent for participation. All those agreeing to participate underwent full-body serial 3D external scans during non-operative treatment or surgical correction. The previous described clinical parameters extracted from the patients’ 3D morphology were prospectively collected. Patients were divided in two groups: group I, pediatric patients under 18 years, and group II, adult patients.

At the initial visit, all patients underwent external 3D scanning. In PE patients, non-operative treatment was proposed in those who showed at least partial correction during the first test application of cup suction (vacuum bell). Partial correction was a qualitative evaluation, defined as a reduction of the deepest point of the deformity during the first test using increasing negative pressure to a maximum of 300 mbar. In PC patients, non-operative treatment was proposed in patients with typical chondrogladiolar PC and when pressure for initial correction (PIC) was ≤9 PSI (pounds per square inch). During non-operative treatment, external 3D scanning was carried out monthly in PC and every 4 months in PE patients until complete correction was achieved. In patients undergoing surgery, an external 3D scan was done before and after surgical correction.

To study the correlation between the different indexes, two studies were conducted:

(1) In 42 patients, CT was additionally performed, and correlations were investigated between the HI calculated based on CT images and the EHI calculated with external scanning.
(2) Correlations between the ECI and PE depth were determined in 208 3D scans from all 100 patients included.

For both studies, a Student test was used to determine whether the correlation coefficient “*r*” between the two measurement methods had statistical significance using the following equation:

$$t\;=\;r*\left(n-\text{2}\right)\text{1}/\text{2}/\left(\text{1}-r^{\text{2}}\right)\text{1}/\text{2}.$$

#### Results

In total, 100 patients (children and young adults) with PE or PC undergoing non-operative or surgical treatment were included in the study. Patients were divided into two groups: group I, pediatric patients <18 years old (mean age 12.5 years, range 5–17 years), including 70 patients (50 males and 20 females), 40 with PC and 30 with PE. There were 45 symmetrical deformities and 25 asymmetrical deformities. Group II, adult patients ≥18 years old (mean age, 23.9 years, range 18–60 years), including 30 patients (21 males and 9 females), 23 with PE and 7 with PC. There were 17 symmetrical deformities and 13 asymmetrical deformities.

The *r*-value for the correlation between the HI and EHI was 0.83. We obtained the following values for the HI and EHI: *t* (our cases) = 9.41 and *t* (table) = 2.021. Since the value for *t* (our cases) was greater than the value for *t* (table), we rejected H0, indicating that there was a significant correlation between the HI and EHI (Table 1).

**Table 1:** Correlation between Haller index (HI) and external Haller index (EHI).

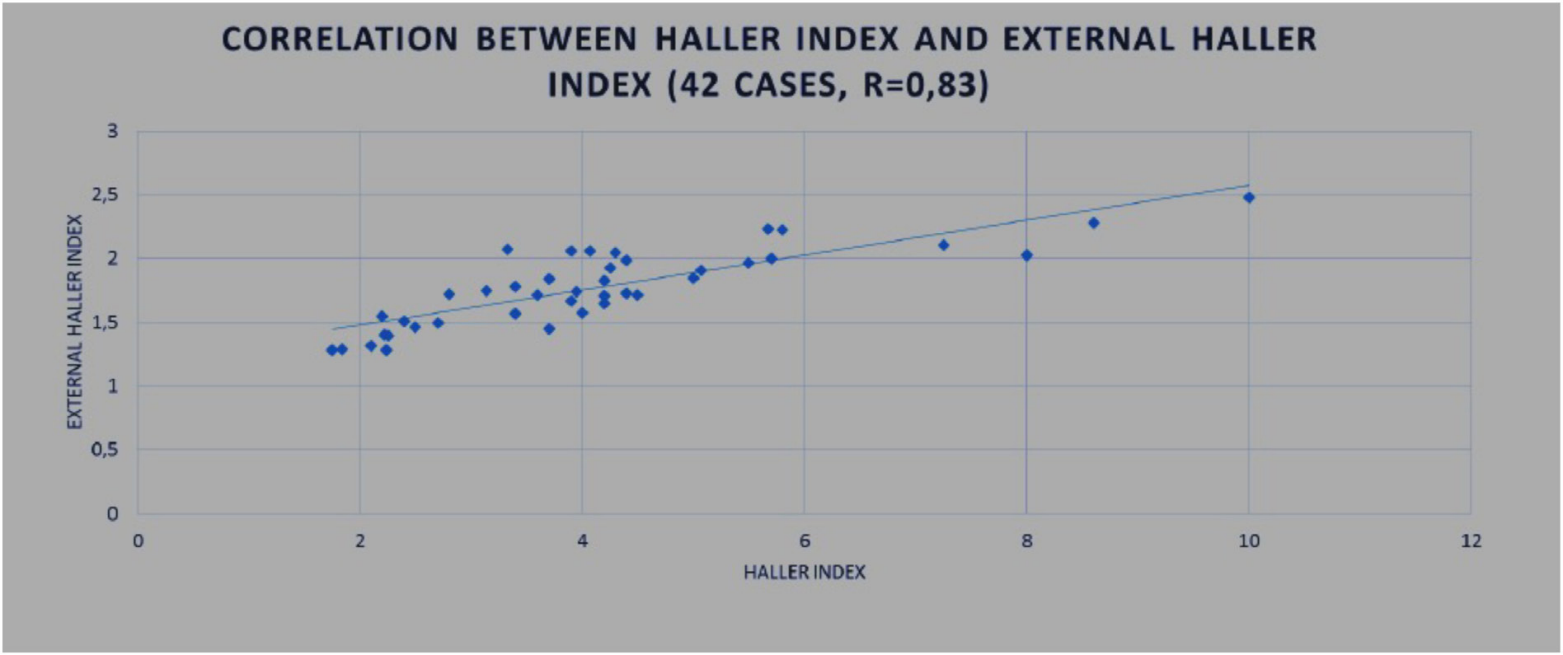
42 patients were evaluated. The following values were obtained for the HI and EHI: *t* (our cases) = 9.41 and *t* (table) = 2.021; *r* = 0.83. A significant correlation was found between the HI and EHI.

In the 208 scans from the 100 patients evaluating correlations between the ECI and PE depth, the *r*-value was 0.89. In this case, the results of the study were as follows: *t* (our cases) = 28.01 and *t* (table) = 1.960. Since the value for *t* (our cases) was greater than the value for *t* (table), we rejected H0, indicating a significant correlation between ECI and PE depth (Table 2).

**Table 2:** Correlation between the external correction index (ECI) and deepest point in pectus excavatum (PE).

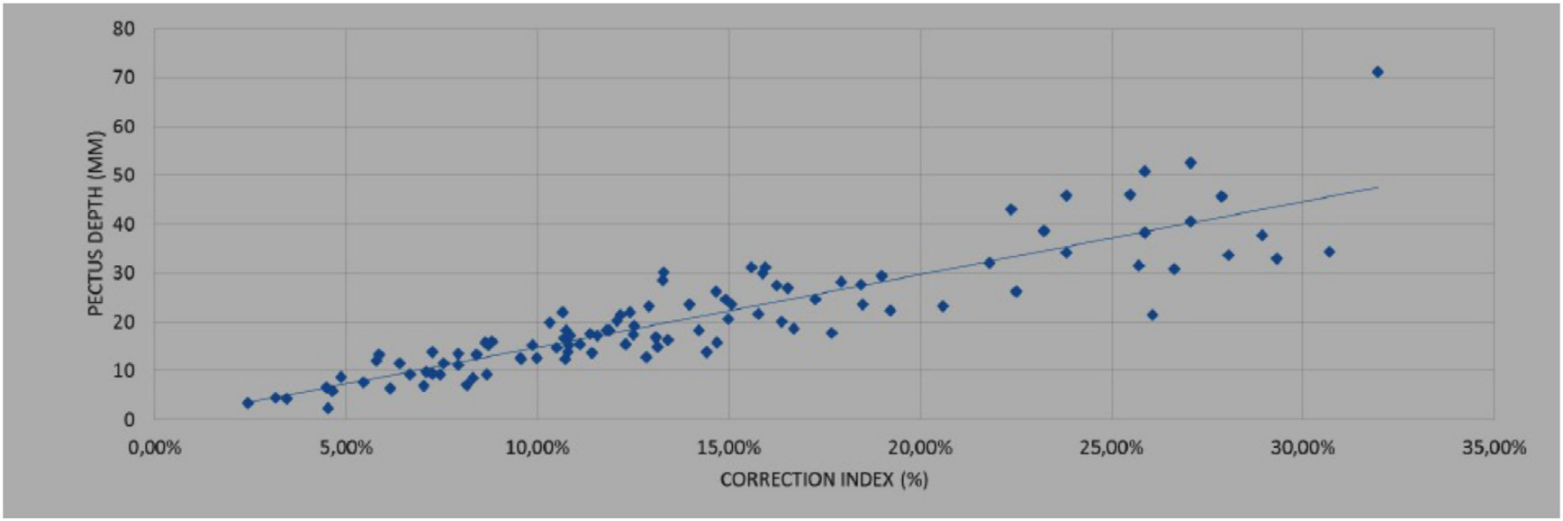
208 3-dimensional scanners were evaluated. The correlation between the ECI and PE depth was *r*: 0.89, with the following values: *t* (our cases) = 28.01 and *t* (table) = 1.960. There was a significant correlation between the ECI and PE depth.

### Discussion

#### Advantages and Limitations

Currently, the standard methods to evaluate the severity of chest wall deformities include radiography, CT scanning, and MRI. These imaging techniques are used to assess the abnormality and calculate several relevant indexes, such as the HI and CI. Both radiography and CT study involve the use of ionizing radiation. Although efforts have been made to reduce radiation dose in CT examinations by single-slice or low-dose techniques, these studies still involve considerable radiation exposure, which limits their suitability when repeated examinations are needed in certain conditions. This is particularly true for the pediatric population, which is more vulnerable to the effects of ionizing radiation.

Magnetic resonance imaging can also be used for chest wall evaluation and it does not involve radiation exposure. Nonetheless, MRI is not as widely available as other imaging modalities, and the examination is longer and more costly, making it a less reasonable option for repeated examinations in milder conditions. Thus, patients with minor or moderate chest deformities are often evaluated using qualitative methods, and may remain without an objective means for diagnostic and follow-up assessment. To fill this gap, external 3D scanning has been developed, a fast, objective assessment tool that does not involve ionizing radiation and can be implemented in the outpatient consultation setting.

In addition to reproducible diagnostic evaluation, external 3D scanning has other advantages. In PC patients who are candidates for non-operative treatment with dynamic compression, the software automatically provides chest measurements needed to customize the brace for individual patients. In the follow-up of these treatments, longitudinal analysis can be used to monitor their effectiveness. Images from serial studies can be superimposed to detect differences in the measures and indexes over time. In addition, measures before and after surgical treatment can be compared. Thanks to the different indexes and measurements obtained by the 3D scan we can also plan reconstructive surgery in PE. It allows calculating the number of bars required, and although this is a future task, we believe it can help to design the bars prior to surgery.

In the present study, we found a significant correlation between the investigated indexes measured by CT scanning and those measured by 3D scanning, and we are currently working to further characterize the external indexes. Although additional studies are needed to determine the correlations associated with all the external measures and indexes, we found that the external 3D scanning data is useful to plan strategies for corrective PE surgery by locating the optimal position for Nuss bar placement and the number of bars required (external banana and Titanic indexes). As external 3D scanning is easily reproducible, we are working on studies involving other centers.

Determining the treatment approach to use in patients with mixed deformities can be a challenge, particularly in non-operative treatments using dynamic compression devices for PC vacuum bell suction for PE. The capability for colorimetric evaluation enables objective assessment of the changes occurring in the malformation under treatment and provides an indication of the optimal time point to switch treatment. At the same time, the software provides an evaluation of possible musculoskeletal abnormalities, such as postural and spinal changes, and allows different degrees of scoliosis to be detected. In addition, changes occurring in the spine during treatment can be monitored.

Nevertheless, external 3D scanning also has limitations. Body surface scans strongly rely on the body constitution and this factor may affect the results. The chest measures in obese patients and women (because of the breasts) may differ from those of very thin patients, particularly in PE. Performing two measures reduces this bias. Currently, in severe malformations requiring surgical correction, a complete CT or MRI study cannot be avoided, as it is essential to have information on the status of the bony chest wall, heart, and lungs. However, external 3D scanning can be used to assess the outcome after surgical correction and it is a valuable tool to evaluate the evolution.

In this study, the external 3D scanner was used in both children and adults, but it is limited to children taller than 100 cm. Another current limitation of the scan is evaluation of sternal torsion, although we now working to improve this aspect.

## Conclusion

The new imaging method for evaluating chest wall deformities provided by the OrtenBodyOne scanner is an effective, objective, radiation-free technique that can be use for diagnostic purposes, for monitoring non-operative treatment, and for determining the outcome of surgical correction. Externally measured indexes can be used to evaluate the severity of the deformity without the need for CT scanning. The asymmetry of the deformity can be characterized and the back can be assessed for postural or scoliotic abnormalities. External 3D scanning may be particularly appropriate as a first-line examination for pediatric patients.

## Author Contributions

AL and OT: drafting and acquisition of data analysis. CG and LG: analysis. ML: study conception and design-critical revision.

## Conflict of Interest Statement

The authors declare that the research was conducted in the absence of any commercial or financial relationships that could be construed as a potential conflict of interest.
